# Stress contagion in school: A multiverse analysis of social influence on school-related stress

**DOI:** 10.1371/journal.pone.0348437

**Published:** 2026-05-04

**Authors:** Björn Högberg, Cristian Bortes

**Affiliations:** 1 Department of Social Work, Umeå University, Umeå, Sweden; 2 Centre for Demographic and Ageing Research (CEDAR), Umeå University, Umeå, Sweden; 3 Department of Social Work, Uppsala University, Uppsala, Sweden; 4 Department of Education and Special Education, University of Gothenburg, Gothenburg, Sweden; University of Glasgow, UNITED KINGDOM OF GREAT BRITAIN AND NORTHERN IRELAND

## Abstract

**Introduction:**

Studies have found that behaviors, beliefs, and emotions can be socially contagious and spread between students. Qualitative research suggests that this may apply to school-related stress as well, but quantitative evidence remains limited and potentially sensitive to methodological choices. This study investigated (1) whether individual students’ school-related stress is influenced by their classmates’ stress, and (2) to what extent estimates of such influence vary depending on different methodological choices.

**Methods:**

Panel data from two cohorts of Swedish secondary school students (51.7% girls; M_age_ wave 1 = 13.0, SD = 0.18; M_age_ wave 2 = 16.0, SD = 0.19) were used. Data were analyzed using multiverse analysis, systematically varying the type of statistical model (unit fixed effects, lagged dependent variable, prospective cohort, and school fixed effects models), measurement of stress (continuous vs. dichotomized), operationalization of classmates’ stress, sample restrictions, and control variables.

**Results:**

Of 9,240 model specifications, approximately 80% yielded positive stress contagion effects. However, these were generally small (standardized effects: 0–0.15; odds ratios: 1–1.2) and mostly non-significant, and the size as well as sign of the effects varied substantially across model types.

**Conclusions:**

The findings suggest that stress appears to be driven primarily by other factors that may cluster within schools or classes but that contagion *per se* is probably less important. Reducing school-related stress requires direct intervention focused on concrete and institutional stressors in school rather than targeting direct interpersonal stress transmission. The overall fragility of the estimated contagion effects underscores the importance of methodological transparency and systematic robustness analyses.

## Introduction

School is consistently ranked as among the most important stressors in adolescents’ lives [1–3] and school-related stress is robustly linked to mental health problems as well as to reduced well-being [4,5]. Research on school-related stress has mostly centered on individual-level factors, such as achievement goals [6], or the teaching or learning environment [7]. However, school is also an important social arena, where students spend several hours per day interacting with classmates. Accordingly, a growing literature has examined *social contagion* (or peer influence) effects on educational outcomes such as motivation [8].

Although students report that stress is endemic, normalized, and talked about openly in school [3,9,10], only two studies have investigated stress contagion in schools. Kärner et al. [11] used high-frequency data on school-related stress among 53 German students in two classrooms, while Kiuru et al. [12] used a two-wave panel of school burnout among 611 students in eight schools in Finland. Both studies found evidence of stress contagion between students, meaning that classmates’ stress predicted individual stress. However, identifying social contagion effects from observational data requires strong assumptions [13], and purported contagion effects may be highly sensitive to arbitrary methodological choices [14].

The aim of the present study was, thus, to investigate [1] whether individual students’ school-related stress is influenced by their classmates’ stress, and [2] to what extent estimates of such influence vary depending on different methodological choices. To this end, we used a two-wave panel on Swedish secondary school students and a multiverse analysis that combines results from thousands of model specifications, varying the choice of control variables, sample characteristics, measurement of the focal outcome and exposure, and type of statistical model.

Multiverse analysis is an approach to empirical analysis in which different combinations of reasonable analytical choices are systematically tested [15]. The motivation is that empirical research involves a range of arbitrary choices at each stage of the research process, from data cleaning, to variable transformations, measurement of key variables, inclusion of control variables, and choice of statistical models. Each choice means that an alternative is disregarded, and the outcomes that would have resulted from alternative choices are not observed. Thus, the final results reported in a paper are only a tiny fraction of the “multiverse” of results that could have been obtained if alternative, but equally reasonable, choices had been made.

Importantly, classical (frequentist) statistics start from the assumption that the true model is known, and that all uncertainty in the estimation – as captured by standard errors, p-values, and confidence intervals – comes from sampling [16]. The goal of multiverse analysis is to quantify the uncertainty generated by the fact that the true model is typically unknown and that many different models are equally reasonable [17,18]. It does so by systematically testing numerous combinations of reasonable analytical choices and summarizing the results in a concise and transparent way.

## Research background

### Conceptual clarifications

Social contagion refers to the spread or transmission of behaviors, emotions, or attitudes through a population, group or network. Social contagion theory is thus a theory of social influence, but with a specific focus: to what degree does the presence of *X* (e.g., school-related stress) in a population influence the probability of *X* in an individual who in some way interacts with this population?

The definition of stress in this study draws on Lazarus’ & Folkman’s [19] transactional model of stress and coping. The model posits that stress reactions arise from individuals’ *appraisals* of a situation. Primary appraisals refer to whether a situation – a potential stressor – is judged to pose a threat to the person’s safety or well-being. Secondary appraisals refer to whether the person assesses that they can cope with the situation, given their available resources. If a situation is deemed both threatening and difficult to cope with, emotional, behavioral, cognitive and/or stress reactions can occur. School-related stress thus refers to stress reactions triggered by situations in school. In qualitative studies, adolescent students largely use the term stress to describe negative experiences (i.e., as *distress*; see [20,21]), and report that school-related stress manifests as worry or rumination, unpleasant emotions and psychosomatic problems [10,22,23].

### Social contagion of school-related stress

A key precondition for stress contagion is that stress reactions are observable. Only if stress reactions can be detected, consciously or unconsciously, by others will they influence other students. Behavioral stress reactions are often readily observable, as in restlessness, pacing, fidgeting, or irritability. Emotional stress reactions may be less visible, but research suggests that emotion recognition among humans tends to be at least moderately accurate among people in the same group [24].

Importantly, qualitative research provides some evidence that stress is often openly displayed in school, and that students can perceive when their classmates experience stress. For instance, Spencer et al. [3] write that American high school students reported “pervasive stress to be the norm” and that ”girls who were not showing signs that they were experiencing high levels of stress” were interpreted as “not working hard enough”. Studies of Swedish secondary students corroborate this picture: Låftman et al. [9] write about “a culture of stress in the school”, with one informant stating that students “get stressed out too much”, while Perming et al. [10] report that most of the students “could give examples of how stress manifests itself in others; for example, they seem to be tense, walk fast” and be “easily irritated or angry”.

Beyond this basic precondition, there are at least three mechanisms through which stress can spread in school. First, classmates’ stress reactions are a potentially useful source of information [25]. If others are stressed about a test, it may indicate that the test is consequential and that there may be negative consequences if one fails. In other words, classmates’ appraisals of the situation, expressed through their stress reactions, may influence a student’s primary appraisal of the same situation as threatening. This first mechanism is a form of object-related contagion, where the object of others’ stress is also the object of my stress. Second, if my classmates are stressed about a school task, I may worry about their well-being, even if I do not feel stress about that specific task. Thus, the classmates themselves can become the object of my stress. This is a form person-related stress transmission [25]. Third, emotional contagion theory [26] posits that emotions can spread directly from person to person. This transmission is largely automatic and unconscious and occurs when the ego mimics the facial expression of others (i.e., mimicry), and this then influences the emotional states of the ego.

## Materials and methods

### Institutional background

Swedish compulsory school lasts 10 years (ages 6–16) and is divided into three stages: elementary school (grades 0–3), middle school (grades 4–6), and lower secondary school (grades 7–9). Our data were collected at the end of middle school (grade 6) and the end of lower secondary school (grade 9). The transition from middle to lower secondary school often involves a change in school and classroom composition. While students generally remain with the same classmates throughout grades 4–6 and grades 7–9, the transition in between can entail enrollment in a new and larger school (e.g., 3–6 classes per grade, compared to 1–3 classes in middle school) where students are redistributed into partly new class configurations and face a more complex school environment. The transition to lower secondary school also implies an intensification of academic demands and associated performance-related stress. School-related stress generally peaks in the final year of compulsory school (grade 9) [27], reflecting both developmental factors and the importance of students’ compulsory school leaving grades for access to upper secondary school.

### Participants

We used data from the Evaluation Through Follow-Up (ETF) project, collected by the University of Gothenburg in collaboration with Statistics Sweden [28,29]. The ETF project has collected nationally representative and longitudinal data on different cohorts of Swedish students since the 1960s. In this study, we used data from the two most recent cohorts with complete compulsory school data: cohorts born in 1998 and 2004 and surveyed in grades 6 (age 12–13) and 9 (age 15–16), with data collected between 2011 and 2020.

Approximately 10 000 students were selected in each cohort through cluster sampling, with schools as the primary sampling unit and all students in the selected schools invited to participate. The sample was drawn based on students’ school registration in grade 3, but the surveys were conducted in grades 6 and 9, meaning some students attended different schools when they were surveyed. In the 1998 cohort, 8,007 students (87%) responded to the grade 6 survey and 4,573 (47%) to the grade 9 survey. In the 2004 cohort, 5,190 students (53%) responded to the grade 6 survey and 2,523 (26%) to the grade 9 survey. All participating students were included in the analysis. Non-participating students were excluded. 51.7% of the participants were girls. The average age was 13.0 year (SD = 0.18) in grade 6 and 16.0 years (SD = 0.19) in grade 9.

We examined whether attrition between waves was related to school-related stress (S3 Text and S4 Table). In unadjusted models, both individual stress and class-level stress in grade 6 weakly predicted non-response in grade 9. However, these associations vanished after adjusting for the basic demographic covariates that are included in the main analyses (see below), indicating that attrition is driven by observed and measured characteristics rather than by stress itself.

### School-related stress

School-related stress is in ETF measured using a single item: “How well do the following statements describe your situation at school? I feel stressed”. Response options were [5] “Always / almost always”, [4] “Often”, [3] “Sometimes”, [2] “Rarely”, and [1] “Never/almost never”. Compared to more comprehensive instruments, single items may have lower reliability and content validity. We nonetheless believe the item was useful in this context, for three reasons. First, as evidenced by qualitative research [9,10,20–23], the key term – (school-related) stress – clearly resonates with students, indicating face validity. In these studies, students also describe the different sources (assessments, expectations, time pressure) and manifestations (physiological, psychosomatic, emotional) of stress in ways that resemble both the generic definition of stress in the transactional model [19] and the different facets of stress captured by more comprehensive psychometric instruments [1]. Second, the item is strongly correlated with measures of psychosomatic (r = 0.47–0.53, depending on grade) and emotional (r = 0.56) problems, as well as with school-related worry (r = 0.51–0.52), indicating criterion validity (see S5 Text and S6 Table). Third, Lindblad et al. [30] showed that a similar global item was highly correlated with both factors extracted from a detailed 11 item stress scale, tested on Swedish adolescents.

### Multiverse analysis: defining the model space

The first step in multiverse analysis is defining the model space, that is, identifying the different model ingredients that result from combining different reasonable analytical decisions [18]. In defining the model space, we drew on previous research on social contagion effects (see below). We only considered model ingredients that were possible given the available data. Additionally, we assumed that the goal of the analysis was to identify causal contagion effects. Correlation between individual and peer outcomes, such as similarities in stress levels among students in a class, can arise from three different processes [13]. (a) Correlated effects: students may be similar because they have similar pre-existing characteristics (homophily/selection) or because they share common environmental exposures (confounding); (b) Simultaneity: the individual student may be affected by his or her peers, but may simultaneously influence these peers (i.e., reverse causation). (c) Social contagion effects: individual students’ outcomes are directly influenced by their peers’ outcomes.

The challenge in research on social contagion is to disentangle the social contagion effect from the other sources generating similarity between individual and group outcomes [13]. Researchers have used different strategies to this end, with the perhaps most notable analytical choice concerning the type of statistical model. Other important choices concern restrictions on the sample, the link function used in the model, the measurement of individual and peer characteristics, and the control variables to be included.

### Defining the estimand

Our target estimand [31] is the average causal effect of classmates’ stress on individual student stress: the expected change in a student’s stress level associated with a one-unit increase in the average stress level of their classmates. The four model types we employ use different sources of identifying variation, but all target this same causal parameter under varying assumptions about correlated effects and simultaneity.

### Type of model

#### Unit fixed effects (FE) model.

Unit-FE models only utilize variation in treatment and outcomes within units between measurement points for estimation. This model estimates the average effect of changes in classmates’ stress on individual stress among students who experience variation in exposure over time. Since the exposure – classmates’ stress – is continuous, virtually all (98.3%) students do experience some variation. By design, unit FE addresses correlated effects – both selection into classrooms and confounding – as long as these are time-invariant, but does not resolve simultaneity, as it cannot distinguish whether changes in classmates’ stress induce changes in individual stress or vice versa. Unit-FE models are, moreover, sensitive to dynamic effects, and can produce biased estimates if past treatments directly affect current outcomes or past outcomes directly affect current treatment [32]. Unit FE models are popular in economics, sociology and political science, with Hanushek et al. [33] and Raabe & Wölfer [34] being examples of studies using this approach to study social contagion.

In this study, the unit-FE model was specified as follows:


$$Stress_{ict}=\;\beta_{\text{1}}StressClass_{ct}+\beta_{\text{2}}X_{ict}+\;\tau_{t}+\;\alpha_{i}+\;\varepsilon_{ict}\;$$

Where the change in stress of student *i* in class *c* and grade *t* between grades 6 and 9 is modelled as a function of the corresponding change in their classmates’ stress (StressClass), a vector of covariates (*X*; described below), a grade fixed effect (τ; capturing all temporal changes that are common to all students), a unit fixed effect (α), and an error term (ε) that is assumed to be uncorrelated with classmates’ stress. The key parameter of the model is $\beta_{\text{1}}$, which captures the contemporaneous effect of (a change in) classmates’ stress on individual stress.

#### Lagged dependent variable (LDV) model.

LDV-models estimate the average effect of classmates’ stress on individual stress by including lagged values of the outcome variable as a control. The lagged outcome adjusts for time-invariant selection and confounding that influence baseline stress, thereby addressing correlated effects that operate through these pathways [35]. It does not, however address time-varying confounding between measurement waves [36]. The model partially mitigates simultaneity by conditioning on pre-existing stress levels, but simultaneity bias remains a concern because classmates’ stress and individual stress are measured concurrently in grade 9. The works of Christakis & Fowler [37] are examples of the use of LDVs in the context of social contagion research.

In this study, the LDV model was specified as follows:


$$Stress_{ict}=\;\beta_{\text{1}}Stress_{ict-\text{1}}+\;\beta_{\text{2}}StressCla{ss}_{ct}+\beta_{\text{3}}X_{ict}+\;\tau_{g}+\;\varepsilon_{ict}\;$$

Where $Stress_{ict-\text{1}}$ is the stress score of student *i* in grade 6. The key parameter is $\beta_{\text{2}}$, which reflects the contemporaneous effect of classmates’ stress on individual stress in grade 9. Since the model does not adjust for past values of classmates’ stress, $\beta_{\text{2}}$ also captures any lagged effects of classmates’ stress that are mediated by current classmates’ stress.

#### Prospective cohort model.

Prospective models estimate the average effect of classmates’ stress on individual stress by using lagged classmates’ stress to predict current individual stress, typically while controlling for lagged individual stress. This temporal ordering – where classmates’ stress is measured before the outcome – directly addresses simultaneity concerns by ensuring that individual stress cannot cause classmates’ stress [38]. The model adjusts for correlated effects to the extent these are captured by baseline individual stress, but remains vulnerable to time-varying confounding between measurement waves and to unobserved selection or confounding not reflected in baseline controls. Prospective models are popular in epidemiology and psychology [39], with examples of such approaches in social contagion research being Alho et al. [40] Mendoza & King [41].

The prospective model was specified as follows:


$$Stress_{ict}=\;\beta_{\text{1}}Stress_{ict-\text{1}}+\;\beta_{\text{2}}StressCla{ss}_{ct-\text{1}}+\beta_{\text{3}}X_{ict-\text{1}}+\;\tau_{g}+\;\varepsilon_{ict}\;$$

Where $Stres{sClass}_{t-\text{1}}$ is the stress score of student i’s classmates in grade 6. The key parameter is $\beta_{\text{2}}$, which reflects the lagged effect of classmates’ stress.

#### School fixed effects (FE) model.

In schools with more than one class, school-FE models estimate the average effect of classmates’ stress on individual stress by comparing students across classes within the same school, thereby exploiting within-school between-class variation. This approach automatically adjusts for all school-level confounding factors (resources, policies, neighborhood characteristics), thereby addressing correlated effects operating at the school level. Selection into classes may also be less problematic than selection into schools, as class assignments are typically determined by administrators. However, school FE does not control for individual-level time-invariant confounding or within-school selection, and does not resolve simultaneity bias as individual and classmates’ stress are measured concurrently. Moreover, if contagion effects are heterogenous across school size, the school-FE estimates may diverge from the average treatment effect since only students in schools with at least two classes will contribute to the estimates. School-FE models have been extensively used to study social contagion in economics [33,42].

The school-FE model was specified as follows:


$$Stress_{ic}=\;\beta_{\text{1}}StressClass_{c}+\beta_{\text{2}}X_{ic}+\;\delta +\;\varepsilon_{ic}\;$$

Where $\delta_{i}$ is the school fixed effects. The school-FE model was fitted as a cross-sectional model on the grade 6 students since the participants in ETF are dispersed into more schools after the transition to the upper stage of compulsory school in grade 9. School fixed effects can in principle be incorporated in a longitudinal model, but a cross-sectional analysis was chosen here since school fixed effects is among the most important tools used for drawing causal conclusions concerning contagion from cross-sectional data [43].

Table 1 summarizes the model types.

**Table 1:** Summary of model types.

*Model type*	*Estimand*	*Variation exploited*	*Addresses*	*Does not address*
Unit FE	Average effect of changes in classmates’ stress on changes in individual stress, among students with variation in exposure	Within-person over time	Time-invariant correlated effects (selection, individual and environmental confounders)	Simultaneity; time-varying selection or confounding
LDV	Average effect of classmates’ stress on individual stress, conditioning on baseline stress	Between-person, conditional on baseline stress	Correlated effects (selection, confounding) captured by baseline stress	Time-varying selection or confounding; simultaneity (concurrent measurement)
Prospective cohort	Average effect of baseline classmates’ stress on future individual stress	Between-person, temporal ordering (lags)	Simultaneity (temporal ordering eliminates reverse causation); correlated effects through baseline controls	Time-invariant selection or confounding not captured by baseline controls; time-varying selection or confounding
School FE	Average effect of classmates’ stress on individual stress among students in multi-class schools	Between-class within schools	School-level correlated effects (confounders, between-school selection)	Individual-level confounders, within-school selection; simultaneity (concurrent measurement)

Note: All models target average treatment effects or ATE-like quantities. Instrumental variable models, which estimate local average treatment effects (LATE) for complier subpopulations, are presented separately in S15 Text, S16 Table, S17 Fig, S18 Fig, and S19 Table.

### Sample restrictions and conditions

#### Focus on ”movers”.

Studies on contextual exposures have focused on “movers”: mobile individuals who change their place of residence, work-place, or similar [44]. Analogously, researchers have argued that contagion effects can be identified by focusing on students who change schools or classes [45,46]. Focusing on movers may protect against unobserved confounding since only information from students who change their peer group is used, and may protect against simultaneity since the focal student’s lagged stress score cannot affect the lagged stress scores of his or her *new* classmates [45]. Focusing on movers is not a distinct type of model, but rather a sample restriction. In this study, we therefore combined this with all models described previously save for the school-FE model, which only uses cross-sectional data. Specifically, we varied the share of old classmates – defined as the share of the student’s grade 9 class that attended his or her grade 6 class – when estimating the different models, from using the full sample (no restriction), < 50% of old classmates, or < 25% of old classmates.

#### Measurement error.

Estimates of social contagion effects are sensitive to measurement error of peer characteristics. Classical (random) measurement error generates attenuation bias, while systematic measurement error may generate either inflated or attenuated estimates. Measurement error may result from the measurement of the focal variable (i.e., stress), which in this case is difficult to fix given the data. It may also result from missing data on classmates’ characteristics [47]. To investigate how sensitive the results were to this form of measurement error, we varied the share of students with complete data on stress in the class, from at least 0% (i.e., no restriction), to > 50%, or > 75%. Like the focus on movers, this concerns a sample restriction, and the variation was applied to all types of models.

### Operationalization of stress and choice of link function

#### Individual stress.

The stress-variable ranges from 1 to 5 and can be analyzed as continuous or dichotomized. In this study, we used both approaches, and when dichotomizing, separated the highest score (“Always / Almost always” stressed) from the other response options.

Although stress is measured on a five-point ordinal scale, we believe that the linear models are justified. First, the response categories represent gradations of stress intensity that plausibly reflect an underlying continuous latent variable (see S7 Text and S8 Fig). Second, the multiverse analysis software developed by Young and Holsteen [18] (*multivrs*) does not support ordered logit models, precluding systematic robustness testing across thousands of models. Third, sensitivity analyses using ordered logit for representative models yielded substantively similar conclusions (S9 Text and S10 Table). Fourth, Brant tests revealed violations of the proportional odds assumption, indicating that standard ordered logit models impose inappropriate parametric constraints. The linear models provides more flexible estimation without requiring parallel slopes across thresholds.

#### Link function.

Linear regression models were used together with the continuous measure of stress and logistic regression models with the dichotomized measure.

#### Classmates’ stress.

The workhorse model in social contagion research is the linear-in-means-model, in which the individual outcome is modelled as a function of the average outcome of his or her classmates. The linear-in-means-model has been criticized as overly simplistic, and was in this study complemented with a maximalist approach: the share of classmates reporting the maximal amount (i.e., “Always / Almost always”) of stress [48]. In both cases, the focal student’s score was removed when estimating this average or share.

### Control variables

The last set of analytical decisions considered in this study is the choice of control variables (the “*X*” in the above-described statistical models). Control variables should block the influence of confounders, but not block mediating pathways through which the treatment exerts its influence. Socio-demographic variables may influence, but are not influenced by, classmates’ stress, and were therefore included in all models. These included student grade level, sex, immigration status, parental education, age, and birth cohort, as well as the class average share of girls, foreign-born students, and students with university-educated parents. We also consistently controlled for school ownership (independent vs. public).

The status of other potential control variables available in ETF is less straight-forward, since they may both influence and be influenced by classmates’ stress. These included teaching practices (teacher-centered, student-centered, or student dominated [49]); the class average cognitive ability, grade point average, and share of students with special educational support (as measures of class achievement); as well as class-average indicators of self-reported social exclusion in school, self-reported academic demands, and achievement goal orientations (performance and mastery goals). We measured these as class averages or shares to align them with the measurement of the focal treatment variable. For the additional control variables, we included every possible combination of four of these variables. We restricted the number of additional variables to four per model, since the number of unique combinations increases exponentially with the number of control variables.

The exact measurement of all covariates, along with summary statistics, is described in S1 Table and S2 Table.

### Summary of the multiverse analysis

In a multiverse analysis, the results are primarily summarized using descriptive statistics, such as the mean, median or range of the estimates, or the share of the estimates that are positive, negative and statistically significant. In addition, Young & Holsteen [18] propose a measure called the robustness ratio:


$$Robustness\;ratio=\;\frac{Mean_{\beta }}{\left((\overset{\text{2}}{\underset{S}{\sigma }}+\;\overset{\text{2}}{\underset{M}{\sigma }}\;)\right)}$$

Where $Mean_{\beta }$ is the average regression coefficient (or odds ratio), $\sigma_{S}$ is the average standard error of the regression coefficients, and $\overset{\text{2}}{\underset{M}{\sigma }}$ is the standard deviation of the combined regression coefficients. A higher mean estimate, combined with a lower sampling uncertainty (as captured by $\sigma_{S}$) or model uncertainty (as captured by $\sigma_{M}$), generates a greater robustness ratio and hence indicate more robust results. The robustness ratio is designed to be analogous to a t-statistic in conventional regression analysis, and Young & Holsteen (2017) suggest as a rule of thumb that robustness ratios larger than 2 can be interpreted as indicating robust results.

Table 2 summarizes the different model specifications. In total, 9,240 different models were estimated. Cognitive ability was only available in grade 6, and grade point average in grade 9, meaning that the number of additional control variables varied between different types of models.

**Table 2:** Summary of analytical decisions.

*Type of model*	*Sample restrictions and conditions*	*Operationalization of stress and choice of link function*	*Number and combinations of control variables*	*Number of models*
Unit FE	Share old classmates: 3 variations OR Share classmates with data: 3 variations	Measure of individual stress and link function: 2 variations AND Measure of classmates’ stress: 2 variations	8 variables (excluding cognitive ability and GPA) = 70 combinations	(3 + 3)*2*2*70 = 1680 models
LDV	Share old classmates: 3 variations OR Share classmates with data: 3 variations	Measure of individual stress and link function: 2 variations AND Measure of classmates’ stress: 2 variations	9 variables (excluding cognitive ability) = 126 combinations	(3 + 3)*2*2*126 = 3024 models
Prospective cohort	Share old classmates: 3 variations OR Share classmates with data: 3 variations	Measure of individual stress and link function: 2 variations AND Measure of classmates’ stress: 2 variations	9 variables (excluding GPA) = 126 combinations	(3 + 3)*2*2*126 = 3024 models
School FE	Share classmates with data: 3 variations	Measure of individual stress and link function: 2 variations AND Measure of classmates’ stress: 2 variations	9 variables (excluding GPA) = 126 combinations	3*2*2*126 = 1512 models

Note: The unit-FE model excluded cognitive ability and GPA since they do not vary over time. In the LDV models, cognitive ability is always included since it is measured in grade 6, before classmates’ stress in grade 9. The prospective and school-FE models exclude GPA since it is measured in grade 9, after classmates’ stress which is measured in grade 6.

All analyses were conducted using Stata v 18. Multiverse analysis used the *multivrs*-program for Stata [18]. Ethical approval for use of ETF data was granted by the Swedish Ethical Review Authority on December 16 2024 (reference number: 2024-07679-01). Data were accessed from 15.09.2025 to 24.02.2026. The authors did not have access to information that could identify individual participants during or after data collection. All participants and their guardians provided written informed consent to the use of their data for research purposes. The study was conducted in accordance with the principles of the Declaration of Helsinki.

## Results

Since the continuous and dichotomized measures of stress and their respective link functions (linear or logistic) are not directly comparable, we present the results from these two sets of models separately. In addition to the pooled results, we also present results stratified by model type since the by far greatest source of variation in estimates stems from the type of model.

**Fig 1:**
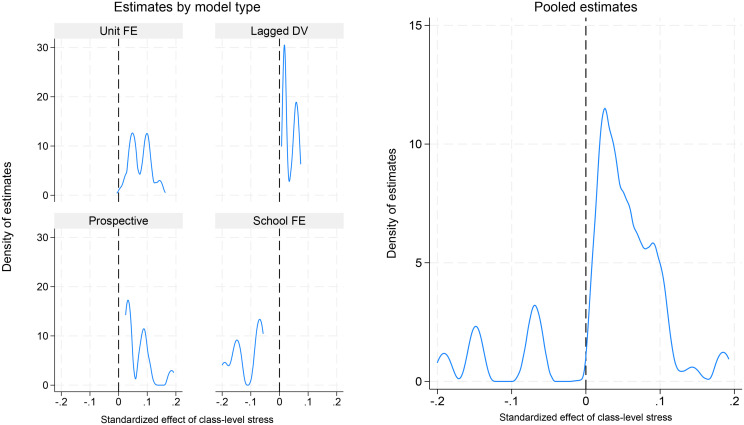
Density plot of standardized effects from linear regression models. Note. Fig shows the distribution of estimates from 6,132 linear regression models.

The distribution of the estimates from the linear models is presented in Fig 1, with standardized effects used to facilitate comparison across models. The pooled estimates vary considerably in size and include both negative and (strongly) positive values, but with a concentration of estimates between 0 and 0.1. Thus, the majority of the estimates are positive but small. The stratified models show that almost all estimates from the unit FE, LDV, and prospective models are positive, while the school-FE models yield consistently negative estimates. Table 3 shows that close to two thirds of the estimates are statistically significant at the 0.05-level, 84% are positive, and 49% are positive and significant. A non-negligible share (16.4%), however, is negative and significant. The robustness ratio is low, 0.37, and well below the suggested cut-off-value of two.

**Table 3:** Distributional, significance testing, and robustness statistics.

	*Linear models*	*Logistic models*
*Distributional statistics (β or OR)*		
p1	−0.19	0.68
p10	−0.07	0.88
P25	0.02	1.03
p50	0.04	1.06
P75	0.07	1.11
P90	0.10	1.15
p99	0.18	1.25
Mean	0.03	1.04
*Significance testing statistics*		
Significance rate	65.5%	27.4%
Positive	83.6%	80.8%
Positive and significant	49.1%	18.2%
Negative	16.5%	19.2%
Negative and significant	16.4%	9.1%
*Robustness statistics*		
Mean	0.03	1.04
Sampling SE	0.02	0.07
Modelling SE	0.08	0.13
Robustness ratio	0.37	0.21
Number of models	4620	4620
Number of observations	1060–17181	488–17181

Note. Abbreviations: *β* = beta coefficient; p = percentile; SE = standard error; OR = odds ratio. Note that the robustness ratio for the logistic models is computed based on log odds, not odds ratios [18].

**Fig 2:**
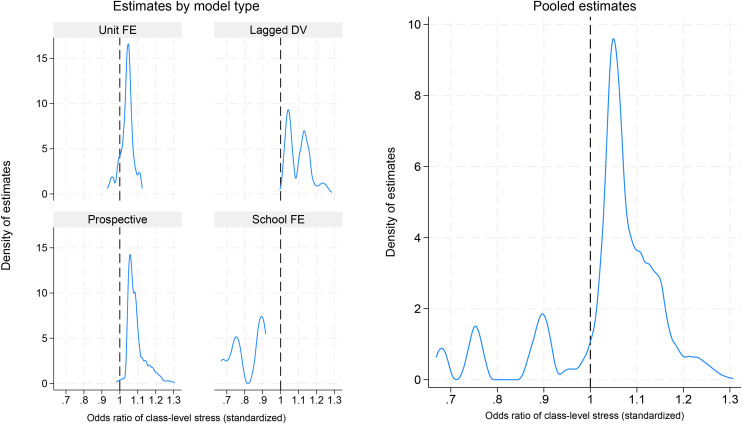
Density plot of odds ratios from logistic regression models. Note. Fig shows the distribution of estimates from 6,132 logistic regression models.

Fig 2 shows corresponding odds ratios from the logistic regression models, with “Always / almost always” stressed as the outcome. The distributions are similar to Fig 1: the pooled estimates vary considerably in size as well as sign, but with a concentration between 1 and 1.2. The stratified models show that the negative estimates come from the school FE models whereas the other model types overwhelmingly yield positive estimates. Table 3 shows that only 27% of the logistic estimates are significant, 81% are positive, and 18% are positive and significant. The robustness ratio is again low, 0.21.

Multiverse analysis also allows for identification of influential model ingredients and analytical choices. Detailed results are presented in S11 Table - S14 Table. Besides the model type – with the negative estimates from the school-FE models diverging from the comparable and largely positive estimates from the other model types – the second greatest source of variation stems from the measurement of stress and the associated link function. Although effect sizes cannot be directly compared across linear and logistic regression models, there are substantially fewer significant estimates from the logistic models. Using stricter definitions of the maximum share of old classmates yields somewhat stronger estimates on average, but also reduces the significance rate due to the smaller sample and more imprecise estimates. Varying the minimum share of classmates with complete data and measurement of classmates’ stress (average vs. maximum), primarily influence the precision, but not the size or sign, of the estimates. The least consequential choice is the choice of control variables, with no single control variable having a substantively important impact on the estimates on average.

## Discussion

Using a two-wave panel on Swedish secondary school students, this study investigated stress contagion in school, specifically: (1) whether individual students’ school-related stress is influenced by their classmates’ stress, and (2) to what extent estimates of such influence vary depending on different methodological choices. Multiverse analysis was used to summarize and compare estimates from different model specifications, varying the choice of control variables, sample characteristics, outcome measure, and type of statistical model.

Results showed that the size as well as the sign of the estimates varied markedly across specifications, with the greatest source of variation stemming from the type of model. The school-FE models yielding consistent negative estimates that contrasted from primarily positive estimates from the other model types. One explanation of this is that the school-FE models rely on a more idiosyncratic source of variation for estimation and may therefore be more sensitive to measurement error. The school-FE models only use within-school variation in stress between classes, meaning that most of the variation in classmates’ stress is effectively disregarded. The remaining variation may be particularly sensitive to measurement error, especially when, as in this case, classmates’ stress was likely measured with some error (see below).

However, even if the school FE-models were to be disregarded for these reasons, the evidence for stress contagion is weak at best (see S20 Fig, S21 Fig, and S22 Table). While the unit-FE, LDV and prospective cohort models mostly yielded positive estimates, less than half were statistically significant, and the effects were consistently small, with standardized coefficients concentrated around 0–0.1 and odds ratios around 1–1.2. All in all, the evidence suggests that stress appears to be driven primarily by other factors that may cluster within schools or classes but that do not primarily spread directly through social contagion. The clustering of stress among classmates may, thus, primarily reflect selection or shared exposure to common stressors – such as performance pressures, academic demands, and expectations – rather than interpersonal transmission *per se*.

These findings have important implications for policy and practice. Schools should not expect that reducing stress among a subset of students will generate cascading effects through peer contagion. Rather than relying on peer multiplier effects, the modest and non-robust contagion effects suggest that prevention requires direct intervention targeted at concrete stressors in school, such as performance-oriented pedagogies [49], assessment practices [50,51], and workload and homework [52]. Relatedly, caution should be exercised about blaming school-related stress on “peer culture,” “school climate” or direct emotional transmission. While peer relationships no doubt matter for adolescent well-being, our results suggest that the proximate drivers of school-related stress are more likely to be institutional or individual-psychological.

The findings also have theoretical implications by suggesting refinements to models of social contagion in educational settings. Behaviors and beliefs that are normative, observable, and socially rewarded, such as achievement goals or educational aspirations, may spread more readily through peer networks [8,34]. Conversely, stress is an internal (cognitive, physiological and emotional) state, and may be less visible and readily transmitted through observation or interaction. Likewise, as a primarily negative phenomenon, stress may also be less normative in most educational contexts, and hence more likely to be actively concealed and less likely to be adopted by others. Moreover, by emphasizing institutional and psychological stressors rather than social transmission, our findings also align with the emphasis on external demands and cognitive appraisals in the transactional model of stress [19]. The weak contagion effects that we observe may thus, from this perspective, reflect the fundamentally individual nature of stress appraisal and coping, even in social and relational contexts such as classrooms.

Another interpretation is that the average effects reported in this study masks important heterogeneity across contexts. Qualitative research suggesting stress contagion in school has been conducted in high-achieving schools and/or focused primarily on girls [3,9,10], and school-related stress may be more normative and visible in such contexts. Nonetheless, our population-level findings caution against generalizing from anecdotal observations of stress contagion among high-achieving girls to sweeping claims about stress dynamics as such.

### Limitations

There are data limitations that restrict the veracity and generalizability of our results. First, stress was measured using a single item, restricting reliability and content validity and increasing measurement error. The importance of measurement error may be compounded when aggregating responses to the class level. Second, there were missing data on classmates, especially in grade 9, which could further compound measurement error. Third, we did not have sociometric data. If students are influenced more by close friends than by the overall class, contagion effects will likely be underestimated. Moreover, lack of sociometric data precludes the use of social network models used to disentangle selection from influence effects. Fourth, the three-year gap between measurements is substantial relative to the likely timescale of stress contagion. In the LDV models, using lagged stress measured three years prior to the outcome may inadequately capture time-varying confounding or selection, although the direction of this form of bias cannot be ascertained *á priori*. The estimates of the prospective models may be attenuated if the true effects weaken over time, while the long interval may introduce noise from intervening factors in the unit FE models; in both cases likely drawing the estimates towards the null. The modest effect sizes we observe may, thus, partly reflect attenuation due to temporal distance. More, or more proximately spaced, measurements may have provided greater power to detect contagion effects. Lastly, for practical purposes, we did not cover the full space of plausible models. For instance, apart from the LDV and prospective models, the four types of models included in our multiverse analysis can be combined in almost any way, and the number of combinations of potentially relevant confounders is practically infinite.

## Conclusions

This study used multiverse analysis to investigate whether school-related stress spreads between students. Across 9,240 model specifications, most models found a positive association between classmates’ stress and individual stress. However, the effects were generally modest and non-significant, and their size as well as sign varied across specifications, suggesting a high degree of model dependency.

All in all, the evidence suggests that stress appears to be driven primarily by other factors that may cluster within schools or classes but do not primarily spread directly through social contagion. Thus, reducing school-related stress requires direct intervention targeted at concrete stressors in school rather than relying on peer multiplier effects. Beyond the substantive results, the findings show that one can find significant contagion effects – in fact, about 3,000 of our specifications did – without these effects being very robust, thus underscoring the importance of methodological transparency and systematic approaches to assessing robustness in contagion research.

Future research should build on these insights by using more comprehensive stress measures, denser longitudinal and sociometric data, and exploring effect heterogeneity across contexts and subgroups. Understanding when and for whom stress spreads in school environments, if at all, is important for social contagion theory as well as for school-based health promotion.

## Supporting information

S1 Table Summary statistics. (DOCX)

S2 Table Measurement of all variables. (DOCX)

S3 Text Attrition analyses. (DOCX)

S4 Table Logistic regression models with non-response in grade 9 as the outcome. (DOCX)

S5 Text Validation of stress-indicator. (DOCX)

S6 Table Bivariate correlations between school-related stress and, respectively, emotional problems, psychosomatic problems, and school-related worry. (DOCX)

S7 Text Distribution of stress. (DOCX)

S8 Fig Distribution of responses in the stress-indicator. (DOCX)

S9 Text Ordered logistic regression. (DOCX)

S10 Table Ordered logistic regression models with school-related stress as the outcome. (DOCX)

S11 Table Influential model ingredients for linear regression models: type of model, sample restrictions, measurement of classmates’ stress. (DOCX)

S12 Table Influential model ingredients for linear regression models: control variables. (DOCX)

S13 Table Influential model ingredients for logistic regression models. (DOCX)

S14 Table Influential model ingredients for logistic regression models: control variables. (DOCX)

S15 Text Instrumental variable analyses. (DOCX)

S16 Table Summary of instrumental variable regression models. (DOCX)

S17 Fig Density plot of standardized effects from linear instrumental variable regression models. (DOCX)

S18 Fig Density plot of standardized effects from logistic instrumental variable regression models. (DOCX)

S19 Table Distributional, significance testing, and robustness statistics for instrumental variable regression models. (DOCX)

S20 Fig Density plot of standardized effects from linear regression models, excluding school fixed effects estimates. (DOCX)

S21 Fig Density plot of standardized effects from linear regression models, excluding school fixed effects estimates. (DOCX)

S22 Table Distributional, significance testing, and robustness statistics, excluding school fixed effects estimates. (DOCX)
