# Supplementary material for: Stress contagion in school: A multiverse analysis of social influence on school-related stress
Source: PLoS One. 2026 May 4;21(5):e0348437. doi: 10.1371/journal.pone.0348437 (PMC13138672; doi:10.1371/journal.pone.0348437)
Supplement: S1 Table — (DOCX) [file pone.0348437.s001.docx]

**S1 Table. Summary statistics**

|  | *Mean* | *Standard deviation* | *Min-max* |
| --- | --- | --- | --- |
| School-related stress | 2.85 | 1.19 | 1-5 |
| Class average stress | 2.85 | 0.69 | 1-5 |
| Class share always stressed | 0.110 | 0.179 | 0-1 |
| *Grade level* |  |  |  |
| Grade 6 | 0.632 |  |  |
| Grade 9 | 0.368 |  |  |
| *Sex* |  |  |  |
| Girl | 0.517 |  |  |
| Boy | 0.483 |  |  |
| *Immigration status* |  |  |  |
| Swedish-born & both parents Swedish-born | 0.813 |  |  |
| Swedish-born & one parent foreign-born | 0.082 |  |  |
| Swedish-born & both parents foreign-born | 0.053 |  |  |
| Foreign-born | 0.052 |  |  |
| *Parental education* |  |  |  |
| Compulsory or unknown | 0.063 |  |  |
| Upper secondary | 0.414 |  |  |
| Tertiary | 0.524 |  |  |
| Age | 14 | 1.45 | 11-17 |
| *Birth cohort* |  |  |  |
| 1998 | 0.604 |  |  |
| 2004 | 0.396 |  |  |
| *School ownership* |  |  |  |
| Public | 0.802 |  |  |
| Independent | 0.198 |  |  |
| Class average share girls | 0.517 | 0.214 | 0-1 |
| Class average share foreign-born | 0.052 | 0.105 | 0-1 |
| Class average share university-educated parents | 0.524 | 0.256 | 0-1 |
| *Teaching practices* |  |  |  |
| Teacher-centered | 3.55 | 0.608 | 1-5 |
| Student-centered | 2.87 | 0.698 | 1-5 |
| Student dominated | 3.63 | 0.566 | 1-5 |
| Class average cognitive ability | 65.95 | 8.72 | 22-114 |
| Class average grade point average | 51.58 | 16.63 | 1-100 |
| Class average special educational support | 0.090 | 0.144 | 0-1 |
| Class average social exclusion in school | 1.64 | 0.468 | 1-5 |
| Class average academic demands | 2.23 | 0.462 | 1-5 |
| *Class average achievement goal orientations* |  |  |  |
| Performance goals | 2.78 | 0.540 | 1-5 |
| Mastery goals | 4.11 | 0.430 | 1-5 |
