# Supplementary material for: Stress contagion in school: A multiverse analysis of social influence on school-related stress
Source: PLoS One. 2026 May 4;21(5):e0348437. doi: 10.1371/journal.pone.0348437 (PMC13138672; doi:10.1371/journal.pone.0348437)
Supplement: S2 Table — (DOCX) [file pone.0348437.s002.docx]

**S2 Table. Measurement of all variables**

| *Variable* | *Measurement* |
| --- | --- |
| School-related stress | Question: “How do the following statements describe your situation in school?”  Item: “I feel stressed”  Response options: "Never / almost never" (1), "Often" (2), "Sometimes" (3), "Rarely" (4), "Always / almost always" (5) |
| Class average stress | Average score (1-5) of classmates on school-related stress |
| Class share always stressed | Share of classmates reporting "Always / almost always" (5) on school-related stress |
| Grade level | The expected grade of the student given their age.  Categories: Grade 6 or grade 9 |
| Sex | Student’s legal gender.  Categories: Boy or girl |
| Immigration status | Country of birth of the student and their parents. Categorized into:  Swedish-born & both parents Swedish-born  Swedish-born & one parent foreign-born  Swedish-born & both parents foreign-born  Foreign-born |
| Parental education | Highest attained education of either parent. Categorized into:  Compulsory or unknown (ISCED 0-2, or missing)  Upper secondary (ISCED 3-4)  Tertiary (ISCED 5-8) |
| Age | Year of survey - year of birth. |
| Birth cohort | The birth cohort in focus for the survey.  Categories: 1998 or 2004 |
| School ownership | The organization responsible for the school  Categories: Public or independent |
| Class average share girls | Share of classmates with female legal gender. |
| Class average share foreign-born | Share of classmates born outside Sweden. |
| Class average share university-educated parents | Share of classmates with at least one university-educated parent. |
| Teaching practices | Question: “How often do you work in this particular way in your class? “  Response options: "Never / almost never" (1), "Often" (2), "Sometimes" (3), "Rarely" (4), "Always / almost always" (5)  The following shows which items are used to indicate which type of teaching practice.  Teacher-centered:  The teacher talks most of the time during lessons.  Have tests  Have written homework assignments  Student-centered:  Being involved in planning instruction  Work in groups  Teacher and students discuss things together  Student dominated:  Work individually  Work with large assignments or projects  Look up information independently  The indicators show students’ average score on the included items. |
| Class average cognitive ability | Students are in the grade 6 survey asked to complete a number of mathematical, logical or linguistic tasks aimed at measuring cognitive ability. Students scores on these tasks are totaled, and the indicator shows the average score of the classmates.  Only available in grade 6. |
| Class average grade point average | The sum of the best 16 subject grades in the 9^th^ grade of compulsory school. The highest grade (A) gives 20 points. A grade below pass-level (E) gives 0 points. Transformed into percentile rank (1-100) within the cohort to account for grade inflation over time, and with the percentile scores averaged over classmates.  Only available in grade 9. |
| Class average special educational support | Share of classmates receiving any type of special support in school in the year of the survey. |
| Class average social exclusion in school | Question: “How do the following statements describe your situation in school?”  Items: “I feel excluded in school”.  Response options: "Never" (1), "Often" (2), "Sometimes" (3), "Rarely" (4), "Always" (5)  The indicator shows average score of the classmates on the included items. |
| Class average academic demands | Question: “How do the following statements describe your situation in school?”  Items: “I find it difficult to keep up in lessons”, “I easily give up if I get a difficult task to do in school”, “I need more help than what I get from my teachers”  Response options: "Never" (1), "Often" (2), "Sometimes" (3), "Rarely" (4), "Always" (5)  The indicator shows average score of the classmates on the included items. |
| Class average achievement goal orientations | Question: “How often do you try and do the following things in school?”  Response options: "Never" (1), "Often" (2), "Sometimes" (3), "Rarely" (4), "Always" (5)  The following shows which items are used to indicate which type of achievement goal orientation.  Performance goals:  Learn so that I wont appear to be stupid in front of others  Learn to be better than other pupils in the class  Learn to show my teacher that I am smarter than other pupils  Mastery goals: Learn to understand better  Learn to be smarter  Learn new things  Work hard to learn, even if it is difficult  The indicators show average score of the classmates on the included items. |
