# Supplementary material for: Stress contagion in school: A multiverse analysis of social influence on school-related stress
Source: PLoS One. 2026 May 4;21(5):e0348437. doi: 10.1371/journal.pone.0348437 (PMC13138672; doi:10.1371/journal.pone.0348437)
Supplement: S3 Text — (DOCX) [file pone.0348437.s003.docx]

**S3 Text. Attrition analyses**

In the 1998 cohort, 8,007 students (87%) responded to the grade 6 survey and 4,573 (47%) to the grade 9 survey. In the 2004 cohort, 5,190 students (53%) responded to the grade 6 survey and 2,523 (26%) to the grade 9 survey. To examine the possible consequences of this fairly high non-response rate, we conducted an attrition analysis.

The outcome variable is coded 1 for students who responded to the grade 6 survey but not the grade 9 survey (i.e., the attrition), and 0 for students who responded to both the grade 6 and the grade 9 surveys. We estimated a series of logistic regression models with this variable as the outcome. We investigated whether individual as well as class-level stress in grade 6 predicted non-response in grade 9, but in unadjusted models and in models adjusted for the basic demographic confounders: student grade level, sex, immigration status, parental education, age, and birth cohort, school ownership (independent vs. public), school ownership (independent vs. public), as well as the class average share of girls, foreign-born students, and students with university-educated parents.

In unadjusted models, both individual stress (OR=1.05; p = 0.019) and class-level stress (OR=1.06; p = 0.010) weakly predicted non-reponse when analyzed separately, and only class-level stress predicted non-reponse when both where included in the model simultaneously. In models adjusted for covariates,neither individual nor class-level stress predicted non-response, with p-values ranging from 0.297 to 0.528.
