# Supplementary material for: Stress contagion in school: A multiverse analysis of social influence on school-related stress
Source: PLoS One. 2026 May 4;21(5):e0348437. doi: 10.1371/journal.pone.0348437 (PMC13138672; doi:10.1371/journal.pone.0348437)
Supplement: S4 Table — (DOCX) [file pone.0348437.s004.docx]

**S4 Table. Logistic regression models with non-response in grade 9 as the outcome**

|  | *Unadjusted models* | | | *Adjusted models* | | |
| --- | --- | --- | --- | --- | --- | --- |
|  | *Model 1* | *Model 2* | *Model 3* | *Model 4* | *Model 5* | *Model 6* |
| Individual stress grade 6 | 1.05* |  | 1.04 | 1.02 |  | 1.02 |
|  | [1.01,1.09] |  | [0.99,1.08] | [0.97,1.07] |  | [0.97,1.07] |
| Class-level stress grade 6 |  | 1.06* | 1.05* |  | 0.98 | 0.97 |
|  |  | [1.01,1.10] | [1.00,1.10] |  | [0.93,1.03] | [0.92,1.03] |
| Adjusted for covariates | No | No | No | Yes | Yes | Yes |
| N | 8816 | 8816 | 8816 | 8521 | 8521 | 8521 |

Note. Table shows odds ratios, with 95% confidence intervals in brackets. Individual and class-level stress have been standardized (mean=0; sd=1)
