# Supplementary material for: Stress contagion in school: A multiverse analysis of social influence on school-related stress
Source: PLoS One. 2026 May 4;21(5):e0348437. doi: 10.1371/journal.pone.0348437 (PMC13138672; doi:10.1371/journal.pone.0348437)
Supplement: S5 Text — (DOCX) [file pone.0348437.s005.docx]

**S5 Text. Validation of stress-indicator**

Worry in school grade 6 is measured by the following three items: I worry about things that happen in school, I worry about tests on homework, I worry about how I am going to pass exams. The items are summed and students’ are assigned the average value for the three items.

Worry in school grade 9 is measured by the item: I worry about things that happen in school.

Psychosomatic and emotional problems are measured in accordance with (Giota & Gustafsson, 2017):

Psychosomatic problems = During the last six months, have you experienced … Difficulties concentrating, Difficulties sleeping, Headache, Stomach ache, Felt tense, Poor appetite, Felt sad and Felt giddy.

Emotional problems = During the last six months, have you … Felt irritated, Felt nervous, Felt down, Conflicts with peers and Withdrawn from peers.

Note that data on psychosomatic and emotional problems are not available in school year 6 for the 1998 cohort.

Reference:

Giota, J., & Gustafsson, J.-E. (2017). Perceived Demands of Schooling, Stress and Mental Health: Changes from Grade 6 to Grade 9 as a Function of Gender and Cognitive Ability. *Stress and Health*, *33*(3), 253-266. https://doi.org/doi:10.1002/smi.2693
