# Supplementary material for: Stress contagion in school: A multiverse analysis of social influence on school-related stress
Source: PLoS One. 2026 May 4;21(5):e0348437. doi: 10.1371/journal.pone.0348437 (PMC13138672; doi:10.1371/journal.pone.0348437)
Supplement: S6 Table — (DOCX) [file pone.0348437.s006.docx]

**S6 Table. Bivariate correlations between school-related stress and, respectively, emotional problems, psychosomatic problems, and school-related worry**

|  | Emotional problems | Psychosomatic problems | School-related worry |
| --- | --- | --- | --- |
| Stress grade 6 | 0.556 | 0.475 | 0.513 |
| Stress grade 9 | 0.560 | 0.531 | 0.523 |
