# Supplementary material for: Stress contagion in school: A multiverse analysis of social influence on school-related stress
Source: PLoS One. 2026 May 4;21(5):e0348437. doi: 10.1371/journal.pone.0348437 (PMC13138672; doi:10.1371/journal.pone.0348437)
Supplement: S7 Text — (DOCX) [file pone.0348437.s007.docx]

**S7 Text. Distribution of stress**

As shown in Fig D1, all five response categories are well-represented at both Grade 6 and Grade 9, with no category containing fewer than 5% of responses. There are no severe floor or ceiling effects: the responses are not bunched at the extremes but show meaningful variation across the full scale. In grade 6, responses cluster around the middle categories (2 and 3), while in grade 9, responses are more evenly distributed across categories 2-5. This pattern indicates that students are using the full range of the scale in a meaningful way, and that the ordered categories plausibly reflect gradations along an underlying continuum of stress. The absence of severe bunching or gaps (e.g., a U-shaped pattern) in the distribution, combined with the reasonable spread across categories, suggests that treating stress as a continuous variable in linear models provides a reasonable approximation of the underlying relationships (cf. Angrist & Pischke (2009)).

Reference:

Angrist, J. D., & Pischke, J. S. (2009). *Mostly harmless econometrics: an empiricist’s companion*. Princeton University Press.
