# Supplementary material for: Stress contagion in school: A multiverse analysis of social influence on school-related stress
Source: PLoS One. 2026 May 4;21(5):e0348437. doi: 10.1371/journal.pone.0348437 (PMC13138672; doi:10.1371/journal.pone.0348437)
Supplement: S9 Text — (DOCX) [file pone.0348437.s009.docx]

**S9 Text. Ordered logistic regression**

Although stress is measured on a five-point ordinal scale (1 = never, 2 = rarely, 3 = sometimes, 4 = often, 5 = always), we treat it as continuous in our primary analyses. To assess the robustness of this decision, we conducted sensitivity analyses using ordered logistic regression for three model types: lagged dependent variable, prospective cohort, and school fixed effects models. We did not replicate the full multiverse with ordered logit because the *multivrs* package in Stata does not support ordered regression commands. Similarly, unit fixed effects models cannot be straightforwardly estimated with ordered outcomes using standard Stata commands.

All models adjust for student grade level, sex, immigration status, parental education, age, and birth cohort, school ownership (independent vs. public), as well as the class average share of girls, foreign-born students, and students with university-educated parents. We also consistently controlled for school ownership (independent vs. public).

The ordered logit and linear specifications yielded substantively similar results across all three model types. In all cases, the estimates contagion effect was had the same sign and similar p-values.
