# Supplementary material for: Stress contagion in school: A multiverse analysis of social influence on school-related stress
Source: PLoS One. 2026 May 4;21(5):e0348437. doi: 10.1371/journal.pone.0348437 (PMC13138672; doi:10.1371/journal.pone.0348437)
Supplement: S10 Table — (DOCX) [file pone.0348437.s010.docx]

**S10 Table. Ordered logistic regression models with school-related stress as the outcome**

|  | *LDV* | | *Prospective* | | *School FE* | |
| --- | --- | --- | --- | --- | --- | --- |
|  | *Ordinal* | *Linear* | *Ordinal* | *Linear* | *Ordinal* | *Linear* |
| Classmates’ average stress | 0.126** | 0.061** | 0.069 | 0.036 | -0.306*** | -0.144*** |
|  | (0.001) | (0.001) | (0.052) | (0.058) | (0.000) | (0.000) |
| N | 8689 | 8689 | 4537 | 4537 | 9994 | 9994 |
