# Supplementary material for: Stress contagion in school: A multiverse analysis of social influence on school-related stress
Source: PLoS One. 2026 May 4;21(5):e0348437. doi: 10.1371/journal.pone.0348437 (PMC13138672; doi:10.1371/journal.pone.0348437)
Supplement: S11 Table — (DOCX) [file pone.0348437.s011.docx]

**S11 Table. Influential model ingredients for linear regression models: type of model, sample restrictions, measurement of classmates’ stress**

|  | Median standardized effect | Share significant estimates | Share positive estimates | Robustness ratios^a^ |
| --- | --- | --- | --- | --- |
| *Type of model* |  |  |  |  |
| Unit fixed effects | 0.074 | 64.9% | 99.5% | 1.53 |
| Lagged dependent variable | 0.033 | 52.1% | 100% | 1.33 |
| Prospective cohort | 0.059 | 61.9% | 100% | 1.38 |
| School fixed effects | -0.111 | 100% | 0.0% | -2.06 |
| *Maximum share old classmates* |  |  |  |  |
| 25% | 0.074 | 49.6% | 100% | - |
| 50% | 0.062 | 50.0% | 100% | - |
| 100% | 0.037 | 71.5% | 77.2% | - |
| *Minimum share classmates with data on stress* |  |  |  |  |
| 0% | 0.045 | 62.3% | 91.1% | - |
| 50% | 0.035 | 75.4% | 71.8% | - |
| 75% | 0.035 | 63.7% | 71.4% | - |
| *Measure of classmates’ stress* |  |  |  |  |
| Class average stress | 0.052 | 65.6% | 83.6% | - |
| Class share maximum stress | 0.039 | 65.3% | 83.4% | - |

Note. The maximum share of old classmates is not varied in the school-FE models, since the school-FE model is cross-sectional; hence the on average larger estimates and greater share of significant and positive estimates in the rows showing values for variation in the maximum share of old classmates.

^a^ Separate robustness ratios are computed for model types due to the great influence of model types on the overall variation of the estimates.
