# Supplementary material for: Stress contagion in school: A multiverse analysis of social influence on school-related stress
Source: PLoS One. 2026 May 4;21(5):e0348437. doi: 10.1371/journal.pone.0348437 (PMC13138672; doi:10.1371/journal.pone.0348437)
Supplement: S12 Table — (DOCX) [file pone.0348437.s012.docx]

**S12 Table. Influential model ingredients for linear regression models: control variables**

|  | Mean difference in standardized effect | Mean difference in share significant estimates | Mean difference in share positive estimates |
| --- | --- | --- | --- |
| *Control variables* |  |  |  |
| Teaching practices: teacher-centered | 0.002 | -1.4% | 0.5% |
| Teaching practices: student-centered | 0.002 | -2.1% | 0.8% |
| Teaching practices: student-dominated | 0.002 | 0.2% | 0.7% |
| Cognitive ability | 0.001 | 11.4% | 0.1% |
| Grade point average | -0.004 | -2.9% | 0.1% |
| Special education needs | -0.003 | 1.0% | 0.7% |
| Social exclusion | 0.001 | -2.2% | 0.7% |
| Academic demands | -0.002 | -2.4% | 0.5% |
| Performance goal orientation | 0.003 | 1.9% | 0.5% |
| Mastery goal orientation | 0.001 | 0.1% | 0.7% |

Note. Table only shows results for control variables that are varied across model specifications.
