# Supplementary material for: Stress contagion in school: A multiverse analysis of social influence on school-related stress
Source: PLoS One. 2026 May 4;21(5):e0348437. doi: 10.1371/journal.pone.0348437 (PMC13138672; doi:10.1371/journal.pone.0348437)
Supplement: S13 Table — (DOCX) [file pone.0348437.s013.docx]

**S13 Table. Influential model ingredients for logistic regression models**

|  | Median odds ratio | Share significant estimates | Share positive estimates | Robustness ratio^a^ |
| --- | --- | --- | --- | --- |
| *Type of model* |  |  |  |  |
| Unit fixed effects | 1.043 | 25.0% | 85.7% | 0.68 |
| Lagged dependent variable | 1.108 | 36.4% | 99.9% | 0.77 |
| Prospective cohort | 1.079 | 5.4% | 99.4% | 1.03 |
| School fixed effects | 0.819 | 55.8% | 0.0% | -1.64 |
| *Maximum share old classmates* |  |  |  |  |
| 25% | 1.101 | 2.9% | 95.6% | - |
| 50% | 1.114 | 13.5% | 99.8% | - |
| 100% | 1.056 | 30.4% | 1 % | - |
| *Minimum share classmates with data on stress* |  |  |  |  |
| 0% | 1.055 | 44.3% | 71.9% | - |
| 50% | 1.049 | 32.2% | 71.9% | - |
| 75% | 1.050 | 30.9% | 30.9% | - |
| *Measure of classmates’ stress* |  |  |  |  |
| Class average stress | 1.060 | 46.1% | 80.2% | - |
| Class share maximum stress | 1.049 | 8.6% | 81.4% | - |

Note. The maximum share of old classmates is not varied in the school-FE models, since the school-FE model is cross-sectional; hence the on average larger estimates and greater share of significant and positive estimates in the rows showing values for variation in the maximum share of old classmates.

^a^ Separate robustness ratios are computed for model types due to the great influence of model types on the overall variation of the estimates.
