# Supplementary material for: Stress contagion in school: A multiverse analysis of social influence on school-related stress
Source: PLoS One. 2026 May 4;21(5):e0348437. doi: 10.1371/journal.pone.0348437 (PMC13138672; doi:10.1371/journal.pone.0348437)
Supplement: S14 Table — (DOCX) [file pone.0348437.s014.docx]

**S14 Table. Influential model ingredients for logistic regression models: control variables**

|  | Mean difference in odds ratio | Mean difference in share significant estimates | Mean difference in share positive estimates |
| --- | --- | --- | --- |
| *Control variables* |  |  |  |
| Teaching practices: teacher-centered | 0.005 | 3.9% | 0.5% |
| Teaching practices: student-centered | 0.001 | 1.3% | 0.4% |
| Teaching practices: student-dominated | -0.002 | 0.3% | 0.0% |
| Cognitive ability | -0.003 | 0.2% | -0.2% |
| Grade point average | -0.003 | -3.2% | -0.1% |
| Special education needs | -0.001 | -0.3% | 0.2% |
| Social exclusion | 0.001 | 0.9% | -0.1% |
| Academic demands | 0.000 | -3.8% | 0.0% |
| Performance goal orientation | 0.002 | 0.0% | -0.4% |
| Mastery goal orientation | -0.002 | -1.1% | -0.5% |

Note. Table only shows results for control variables that are varied across model specifications.
