# Supplementary material for: Stress contagion in school: A multiverse analysis of social influence on school-related stress
Source: PLoS One. 2026 May 4;21(5):e0348437. doi: 10.1371/journal.pone.0348437 (PMC13138672; doi:10.1371/journal.pone.0348437)
Supplement: S15 Text — (DOCX) [file pone.0348437.s015.docx]

**S15 Text. Instrumental variable analyses**

In instrumental variable regression, one or several instruments are, in a first stage, used to predict the treatment. In the second stage, only the part of the treatment that is predicted by the instruments is used to predict the outcome. The instruments must satisfy two conditions: they must be correlated with the treatment and only affect the outcome through their effect on the treatment. The argument for using instrumental variable regression is that it may circumvent both confounding due to omitted variables and simultaneity. Since the validity of specific instruments are context specific, it is difficult to formulate a generic model that represents the approach as a whole. In this study, two instruments are chosen based on their use in previous research on social contagion: lagged values of classmates’ stress (Hanushek et al., 2003), and the share of girls in the class in grade 9 (Hoxby, 2000).

The instrumental variable regression model was specified as follows:

First stage: $StressCla{ss}_{ct}= \beta_{1}Stress_{ict-1}+ \beta_{2}StressCla{ss}_{ct-1}+\beta_{3}GirlsCla{ss}_{ct}+\beta_{4}X_{ict-1}+ \varepsilon_{ict}$

Second stage: $Stress_{ict}= \beta_{1}Stress_{ict-1}+ \beta_{2}\hat{StressCla{ss}_{ct}}+\beta_{3}X_{ict}+ \tau_{g}+ \alpha_{i}+ \varepsilon_{ict}$

Where $StressCla{ss}_{ct}$ is classmates’ stress in grade 9, $StressCla{ss}_{ct-1}$ is classmates’ stress in grade 6, $\hat{StressCla{ss}_{ct}}$ is the predicted values from the first stage regression, and $\beta_{2}$ is the focal parameter.

Instrumental variable (IV) regression models are presented separately in the supplementary materials since these models do not estimate ATE-like estimands. Instead, IV models estimate local average treatment effects (LATE), that is, effects for “compliers”: the individuals that are affected by the instrument in question (or more technically: the effect of the variation in the treatment that is caused by the instrument). The subpopulation of compliers may not be representative of the full population; hence the local in local average treatment effects.

Figs G1 and G2 shows that the IV regression models yield very varied estimates, ranging from clearly negative to in some cases very strongly positive, but with a concentration between -0.05 and 0.1 (linear) and 0.9 and 1.4 (logistic), indicating null or modestly positive effects. The estimates are very imprecise, however, and Table G2 shows that none are statistically significant. The robustness ratios are correspondingly low.

References:

Hanushek, E. A., Kain, J. F., Markman, J. M., & Rivkin, S. G. (2003). Does peer ability affect student achievement? *Journal of Applied Econometrics*, *18*(5), 527-544. https://doi.org/https://doi.org/10.1002/jae.741

Hoxby, C. M. (2000). Peer effects in the classroom: Learning from gender and race variation. National Bureau of Economic Research. Cambridge, Mass., USA.
