# Supplementary material for: Stress contagion in school: A multiverse analysis of social influence on school-related stress
Source: PLoS One. 2026 May 4;21(5):e0348437. doi: 10.1371/journal.pone.0348437 (PMC13138672; doi:10.1371/journal.pone.0348437)
Supplement: S16 Table — (DOCX) [file pone.0348437.s016.docx]

**S16 Table. Summary of instrumental variable regression models**

| *Type of model* | *Sample restrictions and conditions* | *Operationalization of stress and choice of link function* | *Number and combinations of control variables* | *Number of models* |
| --- | --- | --- | --- | --- |
| Instrumental variable model | Share old classmates: 3 variations  OR  Share classmates with data: 3 variations | Measure of individual stress and link function: 2 variations  AND  Measure of classmates’ stress: 2 variations | 9 variables (excluding cognitive ability)  =  126 combinations | (3+3)*2*2*126  = 3024 models |
