# Supplementary material for: Stress contagion in school: A multiverse analysis of social influence on school-related stress
Source: PLoS One. 2026 May 4;21(5):e0348437. doi: 10.1371/journal.pone.0348437 (PMC13138672; doi:10.1371/journal.pone.0348437)
Supplement: S18 Fig — (DOCX) [file pone.0348437.s018.docx]

**S18 Fig. Density plot of standardized effects from logistic instrumental variable regression models**
