# Supplementary material for: Stress contagion in school: A multiverse analysis of social influence on school-related stress
Source: PLoS One. 2026 May 4;21(5):e0348437. doi: 10.1371/journal.pone.0348437 (PMC13138672; doi:10.1371/journal.pone.0348437)
Supplement: S19 Table — (DOCX) [file pone.0348437.s019.docx]

**S19 Table. Distributional, significance testing, and robustness statistics for instrumental variable regression models**

|  | *Linear models* | *Logistic models* |
| --- | --- | --- |
| *Distributional statistics (β or OR)* |  |  |
| p1 | -0.11 | 0.44 |
| p10 | -0.01 | 0.56 |
| P25 | 0.03 | 0.79 |
| p50 | 0.07 | 1.10 |
| P75 | 0.21 | 1.29 |
| P90 | 0.29 | 1.67 |
| p99 | 0.71 | 2.35 |
| Mean | 0.13 | 1.11 |
| *Significance testing statistics* |  |  |
| Significance rate | 0 % | 0 % |
| Positive | 93.5 % | 65.1 % |
| Positive and significant | 0 % | 0 % |
| Negative | 6.5 % | 34.9 % |
| Negative and significant | 0 % | 0 % |
| *Robustness statistics* |  |  |
| Mean | 0.13 | 1.11 |
| Sampling SE | 0.15 | 0.59 |
| Modelling SE | 0.34 | 0.40 |
| Robustness ratio | 0.36 | 0.06 |
| Number of models | 1512 | 1512 |
| Number of observations | 935 – 8857 | 488 – 17181 |

Note. Abbreviations: *β* = beta coefficient; p = percentile; SE = standard error; OR = odds ratio. Note that the robustness ratio for the logistic models is computed based on log odds, not odds ratios (Young & Holsteen, 2017).
