# Supplementary material for: Stress contagion in school: A multiverse analysis of social influence on school-related stress
Source: PLoS One. 2026 May 4;21(5):e0348437. doi: 10.1371/journal.pone.0348437 (PMC13138672; doi:10.1371/journal.pone.0348437)
Supplement: S22 Table — (DOCX) [file pone.0348437.s022.docx]

**S22 Table. Distributional, significance testing, and robustness statistics, excluding school fixed effects estimates**

|  | *Linear models* | *Logistic models* |
| --- | --- | --- |
| *Distributional statistics (β or OR)* |  |  |
| p1 | 0.01 | 0.96 |
| p10 | 0.02 | 1.03 |
| P25 | 0.03 | 1.04 |
| p50 | 0.05 | 1.07 |
| P75 | 0.06 | 1.2 |
| P90 | 0.10 | 1.16 |
| p99 | 0.19 | 1.26 |
| Mean | 0.06 | 1.08 |
| *Significance testing statistics* |  |  |
| Significance rate | 58.7 % | 21.8 % |
| Positive | 99.9 % | 96.6 % |
| Positive and significant | 58.7 % | 21.8 % |
| Negative | 0.1 % | 3.4 % |
| Negative and significant | 0 % | 0 % |
| *Robustness statistics* |  |  |
| Mean | 0.06 | 1. 08 |
| Sampling SE | 0.02 | 0.07 |
| Modelling SE | 0.04 | 0.08 |
| Robustness ratio | 1.28 | 0.93 |
| Number of models | 3864 | 3864 |
| Number of observations | 1060 – 17181 | 488 – 17181 |

Note. Abbreviations: *β* = beta coefficient; p = percentile; SE = standard error; OR = odds ratio. Note that the robustness ratio for the logistic models is computed based on log odds, not odds ratios [18].
